# Eriodictyol suppresses the malignant progression of colorectal cancer by downregulating tissue specific transplantation antigen P35B (TSTA3) expression to restrain fucosylation

**DOI:** 10.1080/21655979.2022.2039485

**Published:** 2022-02-19

**Authors:** Hua Huang, Yun He, Youran Li, Mingjia Gu, Minna Wu, Lijiang Ji

**Affiliations:** a Department of Anorectal, Changshu Hospital Affiliated to Nanjing University of Chinese Medicine, Changshu, Jiangsu Province, China; b Department of Oncology, Changshu Hospital Affiliated to Nanjing University of Chinese Medicine, Changshu, Jiangsu Province, China; c Department of Anorectal, Affiliated Hospital of Nanjing University of Chinese Medicine, Nanjing, Jiangsu Province, China; d Department of Nephrology, Changshu Hospital Affiliated to Nanjing University of Chinese Medicine, Changshu, Jiangsu Province, China

**Keywords:** Eriodictyol, colorectal cancer, TSTA3, fucosylation

## Abstract

Eriodictyol is a natural flavonoid with many pharmacological effects, such as anti-oxidation, anti-inflammation, anti-tumor, and neuroprotection. Besides, it has been reported that flavonoids play an important role in protein glycosylation. The fucosylation structure is closely associated with processes of various tumor metastases. TSTA3 is involved in the de novo synthesis and can convert cellular GDP-D-mannose into GDP-L-fucose. It was predicted on the STITCH database that eriodictyol interacted with TSTA3. In addition, literature has confirmed that TSTA3 is upregulated in CRC and can regulate the proliferation and migration of breast cancer cells. Herein, the precise effects of eriodictyol on the clone-forming, proliferative, migratory and invasive abilities of CRC cells as well as EMT process were assessed. Moreover, the correlation among eriodictyol, TSTA3, and fucosylation in these malignant behaviors of CRC cells was evaluated, in order to elucidate the underlying mechanism. The current work discovered that eriodictyol inhibited the viability, clone-formation, proliferation, migration, invasion, and EMT of CRC cells, and that these inhibitory effects of eriodictyol on the malignant behavior of CRC cells were reversed by TSTA3 overexpression. Additionally, eriodictyol suppresses fucosylation by downregulating the TSTA3 expression. Results confirmed that fucosylation inhibitor (2-F-Fuc) inhibited clone formation, proliferation, migration, invasion, as well as EMT of CRC cells and eriodictyol treatment further reinforced the suppressing effects of 2-F-Fuc on the malignant behavior of CRC cells. We conclude that eriodictyol suppresses the clone-forming, proliferative, migrative and invasive abilities of CRC cells as well as represses the EMT process by downregulating TSTA3 expression to restrain fucosylation.

## Graphical Abstract

**Figure:**
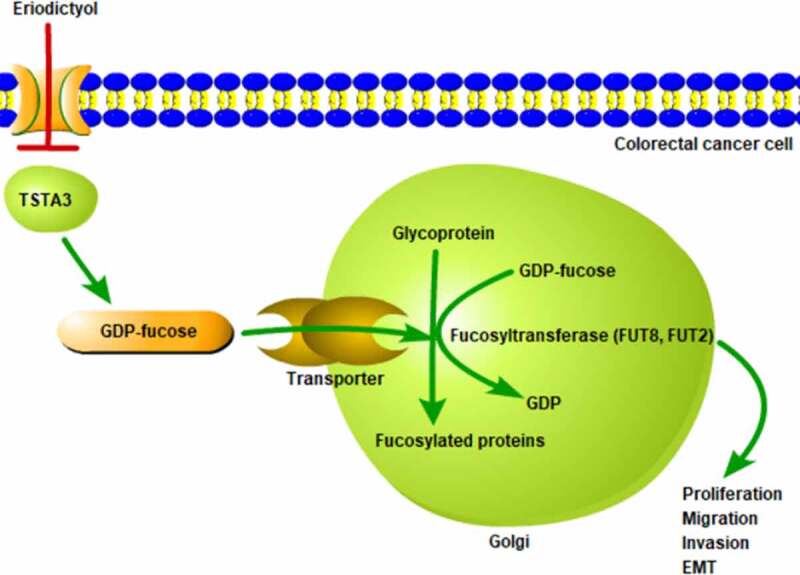

## Introduction

Colorectal cancer (CRC) has a high prevalence worldwide. The morbidity and mortality of CRC are increasing rapidly and maintain an upward trend in the past 5–10 years, largely attributed to dietary changes, aging population, as well as environment pollution [1,2]. According to the global cancer statistics, CRC is the third most commonly diagnosed cancer (10.0% of the total cases) and the second leading cause of cancer-related deaths (9.4% of the total cancer deaths) in 2020 [3]. Tumor metastasis mainly results in treatment failure and cancer-related deaths [4]. Despite the fact that systemic therapies for metastatic CRC have made progress, the prognosis of patients suffering from metastatic CRC still remains poor, and the 5-year survival rate is extremely low [5]. Therefore, in-depth study of the molecular mechanisms underlying CRC occurrence and development will contribute to the exploration of potential therapeutic targets for CRC and improvement of patient prognosis.

Recently, some natural products have been reported to overcome tumor growth and metastasis, including flavonoids that are abundant in plants and foods. Besides, flavonoids are also one of the active ingredients in numerous traditional Chinese herbal medicines [6,7]. Flavonoids have attracted wide attention due to their extensive biological activity, high efficiency, low toxicity, as well as good prevention and cure effects on various types of tumors [8,9]. Eriodictyol is a natural flavonoid with many pharmacological effects, such as anti-oxidation, anti-inflammation, anti-tumor, and neuroprotection [10,11]. Studies have proven that eriodictyol can inhibit proliferation and metastasis of glioma cells [12] and nasopharyngeal cancer cells [13]. Nevertheless, the biological effects of eriodictyol on the malignant development of CRC have not been fully understood until now.

As a vital protein functional modification, protein glycosylation plays an indispensable role in cell adhesion, receptor activation, tumor invasiveness, and metastasis, inflammatory response, as well as other cellular processes [14,15]. Additionally, it has been reported that flavonoids play important roles in protein glycosylation. For instance, Du et al. [16] indicated that genistein could increase IgG glycosylation to decrease the activity of osteoclasts. Core fucosylation catalyzed by fucosyltransferase is one of the most common protein N-glycosylation modifications [17]. The fucosylation structure is associated with the processes of various tumor metastases [18]. A study proves that rosmarinic acid, a natural flavonoid, can suppress the expression of fucosylated antigens in human skin fibroblasts [19]. However, the functional role of eriodictyol in fucosylation structure needs to be deeply elaborated.

GDP-L-fucose synthase (also known as tissue-specific transplantation antigen P35B, TSTA3) is a key gene for the de novo synthesis of GDP-fucose and is essential for the formation of GDP-fucose [20]. It is predicted in the STITCH database that eriodictyol may interact with TSTA3. Importantly, it has been reported that TSTA3 is up-regulated in CRC [21]. Besides, TSTA3 can regulate the proliferation and migration of breast cancer cells [22].

Herein, this work is formulated to explore the anti-cancer potential of eriodictyol against CRC. More importantly, the correlation among eriodictyol, TSTA3, and fucosylation in the development of CRC was investigated to probe into the underlying mechanism.

## Materials and Methods

### Cell culture

Human colon epithelial cell line (FHC) and human CRC cell line (HCT116) were purchased from American Type Culture Collection (ATCC, VA, USA). Cells were cultured in Dulbecco’s Modified Eagle Medium (DMEM; HyClone, UT, USA) supplemented with 10% fetal bovine serum (Gibco, NY, USA) in a humidified atmosphere of 5% CO_2_ at 37°C.

### Cell treatment

To investigate the specific effects of eriodictyol on the malignant behaviors of CRC cells, HCT116 cells were treated with 0, 50, 100, 200, 400, or 600 μM eriodictyol for 24, 48, 72 h.

To investigate the underlying mechanism, HCT116 cells were treated with 15 μM 2-fluoro-L-fucose (2 F-Fuc; Sigma, MO, USA) and/or 400 μM eriodictyol.

### Cell transfection

TSTA3 overexpression lentivirus (Ov-TSTA3) and corresponding negative control (Ov-NC) were obtained from Genechem (Shanghai, China). HCT116 cells were transfected with Ov-TSTA3 or Ov-NC using Lipofectamine^TM^ 2000 (Invitrogen, CA, USA) according to the manufacturer’s protocol.

### Cell counting kit-8 (CCK-8) assay

Cell viability was evaluated using CCK-8 assay. Briefly, the cells were seeded into a 96-well plate (5 × 10^3^ cells/well) for 24, 48, 72 h incubation. CCK-8 solution (Beyotime, Shanghai, China) was added into each well (10 μl) and then incubated for another 4 h. The absorbance was measured at 450 nm with a microplate reader (Bio-Rad, CA, USA).

### Colony formation assay

After resuspension, HCT116 cells were inoculated in 6-well plates (300 cells per well) and incubated in a complete DMEM medium at 37°C for 10 days. Then, cells were fixed with 4% paraformaldehyde for 30 min and stained with 0.1% crystal violet solution for 20 min at room temperature. Visible colonies (>50 cells) were observed and counted under a microscope (Olympus, Tokyo, Japan).

### 5-Ethynyl-2′-deoxyuridine (EdU) staining

The EdU staining kit (Beyotime, Shanghai, China) was employed to determine the proliferative ability of HCT116 cells. HCT116 cells were fixed with 4% formaldehyde and permeabilized in 0.05% Triton X-100 for 10 min. After washing thrice with PBS, HCT116 cells were incubated with EdU working solution in the dark for 30 min and stained with DAPI in the dark for 15 min. Stained cells were observed and photographed under a fluorescent microscope (magnification, x100; Olympus, Tokyo, Japan).

### Wound healing assay

HCT116 cells were cultured in 6-well plates until they reached 85% confluence. Cells were wounded with a 200 μl pipette tip, and the detached cells were washed twice with PBS. Subsequently, the cells were incubated in serum-free DMEM for 24 h, and the images of the wounds were captured at 0 and 24 h under a microscope (magnification, ×100; Olympus, Tokyo, Japan). Cell migration was quantified by determining the extent (%) of wound healing as follows (0 h scratch area – 24 h scratch area)/0 h scratch area × 100%.

### Transwell invasion assay

Transwell chambers (Corning, NY, USA) precoated with Matrigel (BD Biosciences, CA, USA) were employed to detect cell invasion. Briefly, 1 × 10^5^ cells suspended in a serum-free medium were seeded into the upper chamber, and 600 µl FBS-DMEM was added into the lower chamber. After 48 h incubation, the noninvasive cells were gently removed, and the invaded cells in the lower chamber were fixed with 4% paraformaldehyde and stained with 0.1% crystal violet solution for 20 min at room temperature. Stained cells were photographed and counted under a microscope (magnification, x100; Olympus, Tokyo, Japan).

### Reverse transcription-quantitative polymerase chain reaction (RT-qPCR)

The extraction of total RNA was operated using TRIzol reagent (Invitrogen, CA, USA). The RNA samples (1 µg) were reversely transcribed into cDNA using a reverse transcription kit (Takara, Tokyo, Japan). Subsequently, PCR reactions were carried out with the One-Step SYBR Prime-Script RT-PCR Kit (Takara, Tokyo, Japan) on the ABI 7500 Sequence Detector System (ABI/Perkin Elmer, CA, USA). The PCR thermocycling conditions were as follows: denaturation at 94°C for 5 min, 40 cycles of denaturation at 94°C for 30 s, annealing at 53°C for 30 s and extension at 72°C for 35 s. The relative gene expressions were calculated with the 2^−∆∆Ct^ method [23]. GAPDH served as the internal control. The primer sequences were listed as follows: TSTA3: forward 5′- GGATGCTCCGTGCAACTG −3′; reverse 5ʹ- CGGGTAGGTCGTCTTGTCAG −3ʹ; GAPDH: forward 5ʹ- CCAGGTGGTCTCCTCTGA −3ʹ; reverse 5ʹ- CCGTGTTCCTACCCCCAATG −3ʹ.

### Western blotting analysis

The extraction of total proteins was conducted using a RIPA lysis buffer (Beyotime, Shanghai, China), and a BCA kit (Beyotime, Shanghai, China) was employed to examine protein concentrations. Equal amounts of protein samples were separated by sodium dodecyl sulfate-polyacrylamide gel electrophoresis (SDS-PAGE) and then transferred onto polyvinylidene difluoride (PVDF) membranes (Millipore, MA, USA). After blocking in 5% nonfat milk for 1 h at 37°C, primary antibodies against MMP2 (Abcam, ab181286, 1:1000), MMP9 (Abcam, ab76003, 1:5000), N-cadherin (Abcam, ab76011, 1:5000), β-cadherin (Abcam, ab32572, 1:5000), Vimentin (Abcam, ab92547, 1:5000), E-cadherin (Abcam, ab40772, 1:5000), TSTA3 (Abcam, ab155306, 1:3000) and GAPDH (Abcam, ab181602, 1:10,000) were applied to incubate the membranes at 4 °C overnight. The next day, the incubation of membranes with horseradish peroxidase (HRP)-conjugated secondary antibody (Abcam, ab205718, 1:50,000) lasted 1.5 h. Protein bands were visualized using enhanced chemiluminescence reagents, and protein signals were analyzed with the application of Image J software.

### Enzyme activity assay

The FUT2 and FUT8 enzyme activities were measured using the Glycosyltransferase activity kit (R&D system, MN, USA) according to the manufacturer’s instructions. The kit utilizes a colorimetric assay specific for phosphate-coupled glycosyltransferase reactions to assay the enzyme activities. The activity of FUT2 in the cell lysate was measured by its ability to transfer fucose from GDP-L-Fucose to alpha-lactose (Sigma-Aldrich, MO, USA). The activity of FUT8 in the cell lysate was measured by its ability to hydrolyze the donor substrate GDP-L-Fucose (Santa Cruz, CA, USA). The glycosyltransferase activity was expressed as phosphate concentration equivalents produced per minute (M P/min).

### Statistical analysis

Data from three independent experiments are presented as means ± standard deviation (SD). A one-way analysis of variance (ANOVA) followed by Tukey’s post hoc test was employed for comparisons among multiple groups, and Student’s t-test was utilized for comparisons between the two groups. P < 0.05 represented a statistically significant difference.

## Results

### Eriodictyol treatment inhibits the viability, clone-forming ability, and proliferation of CRC cells

**Figure 1.**
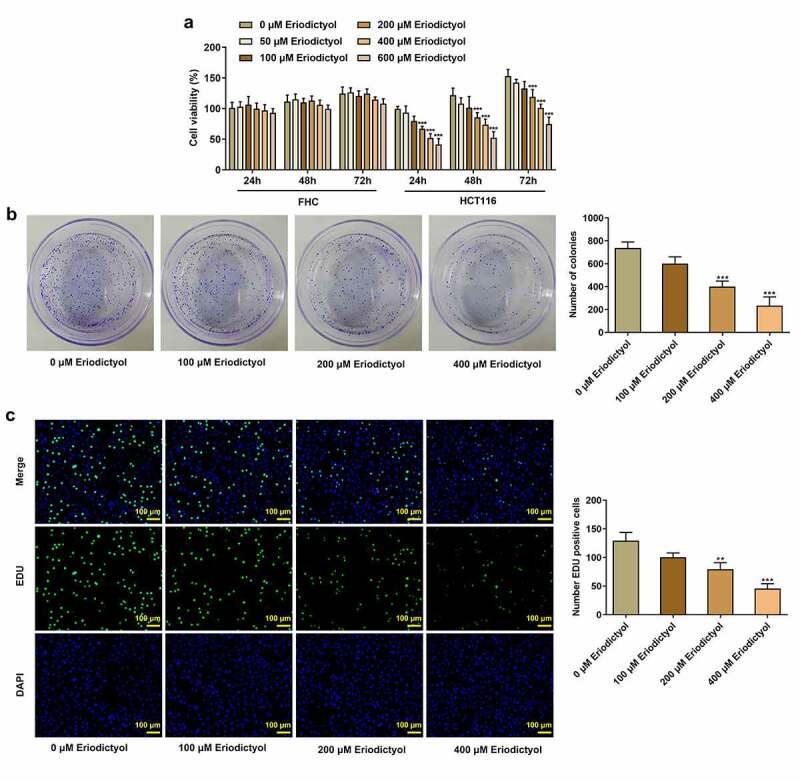
Eriodictyol treatment inhibits the viability, clone-forming ability and proliferation of CRC cells. (a) FHC and HCT116 cells were treated with 0, 50, 100, 200, 400, or 600 μM eriodictyol for 24, 48, 72 h. CCK-8 assay was employed to detect cell viability. (b) HCT116 cells were treated with 0, 100, 200, 400 μM eriodictyol for 48 h. Colony-formation assay was employed to detect the clone-forming ability of CRC cells. (c) HCT116 cells were treated with 0, 100, 200, 400 μM eriodictyol for 48 h. EdU assay was employed to detect CRC cell proliferation. ** p < 0.01, *** p < 0.001 versus 0 μM Eriodictyol.

Firstly, human colon epithelial cell line (FHC) and human CRC cell line (HCT116) were treated with 0, 50, 100, 200, 400, or 600 μM eriodictyol for 24, 48, 72 h to assess the influence of eriodictyol on CRC cell viability. Eriodictyol treatment had no obvious effect on the viability of FHC cells, while eriodictyol significantly inhibited the viability of HCT116 cells in a dose-dependent manner (Figure 1(a)). Then, treatment with 0, 100, 200, 400 μM eriodictyol for 48 h was selected for subsequent experiments according to the results above. Colony-formation and EdU assays indicated that eriodictyol treatment suppressed the clone-forming and proliferative abilities of HCT116 cells (Figure 1(b, c)).

### Eriodictyol treatment suppresses migration, invasion, and EMT of CRC cells

**Figure 2.**
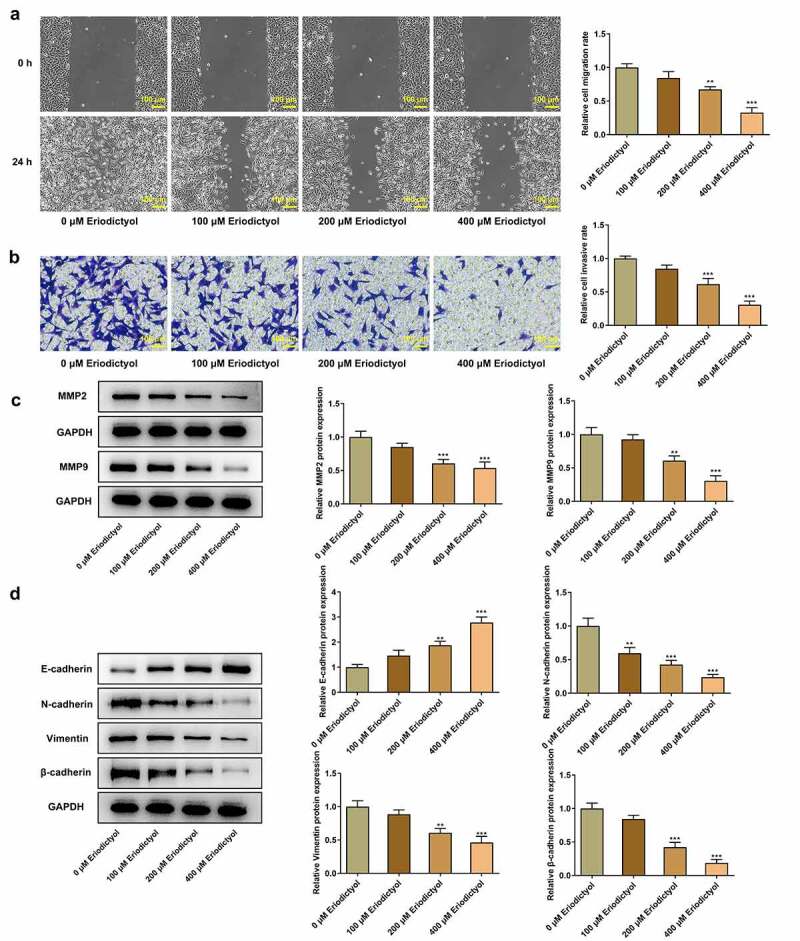
Eriodictyol treatment suppresses migration, invasion, and EMT of CRC cells. HCT116 cells were treated with 0, 100, 200, 400 μM eriodictyol for 48 h. (a) Wound healing assay was employed to detect CRC cell migration. (b) Transwell invasion assay was employed to detect CRC cell invasion. (c) Western blot analysis was employed to detect the levels of MMP2 and MMP9 in CRC cells. (d) Western blot analysis was employed to detect the levels of E-cadherin, N-cadherin, Vimentin, and β-cadherin in CRC cells. ** p < 0.01, *** p < 0.001 versus 0 μM Eriodictyol.

Results from wound healing assay and transwell assay demonstrated that eriodictyol dose-dependently diminished the migrative and invasive properties of HCT116 cells (Figure 2(a, b)). Besides, decreased expressions of MMP2 and MMP9 in HCT116 cells receiving eriodictyol treatment also suggested that eriodictyol could suppress CRC cell migration and invasion (Figure 2(c)). Moreover, elevated E-cadherin expression and reduced expressions of N-cadherin, Vimentin, and β-cadherin in HCT116 cells after eriodictyol treatment indicated that eriodictyol inactivated EMT process of CRC cells (Figure 2(d)).

### Eriodictyol treatment reduces TSTA3 expression in CRC cells

**Figure 3.**
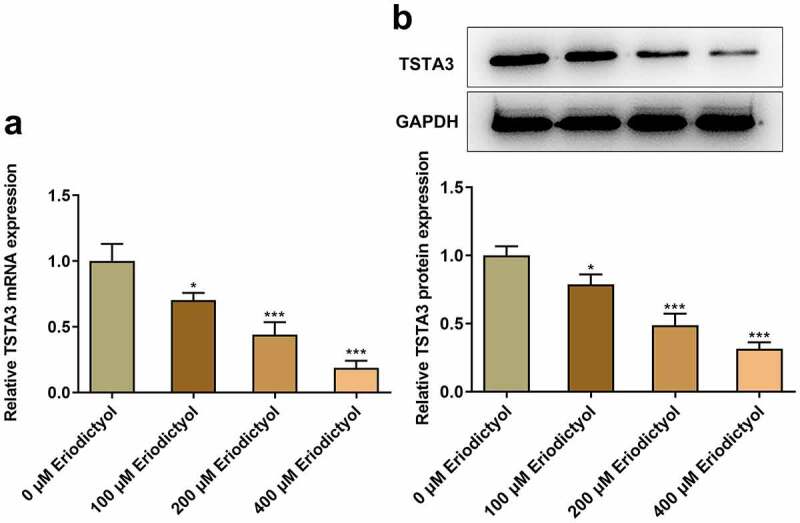
Eriodictyol treatment reduces TSTA3 expression in CRC cells. HCT116 cells were treated with 0, 100, 200, 400 μM eriodictyol for 48 h. (a) RT-qPCR was employed to detect TSTA3 mRNA level in CRC cells. (b) Western blot analysis was employed to detect TSTA3 protein level in CRC cells. * p < 0.05, *** p < 0.001 versus 0 μM Eriodictyol.

Furthermore, eriodictyol associated genes were analyzed on STITCH database, and it was predicted that eriodictyol may interact with TSTA3. Importantly, eriodictyol treatment dose-dependently diminished the mRNA and protein levels of TSTA3 in HCT116 cells (Figure 3(a, b)).

### Overexpression of TSTA3 reverses the inhibitory effects of eriodictyol on the clone-forming and proliferative abilities of CRC cells

**Figure 4.**
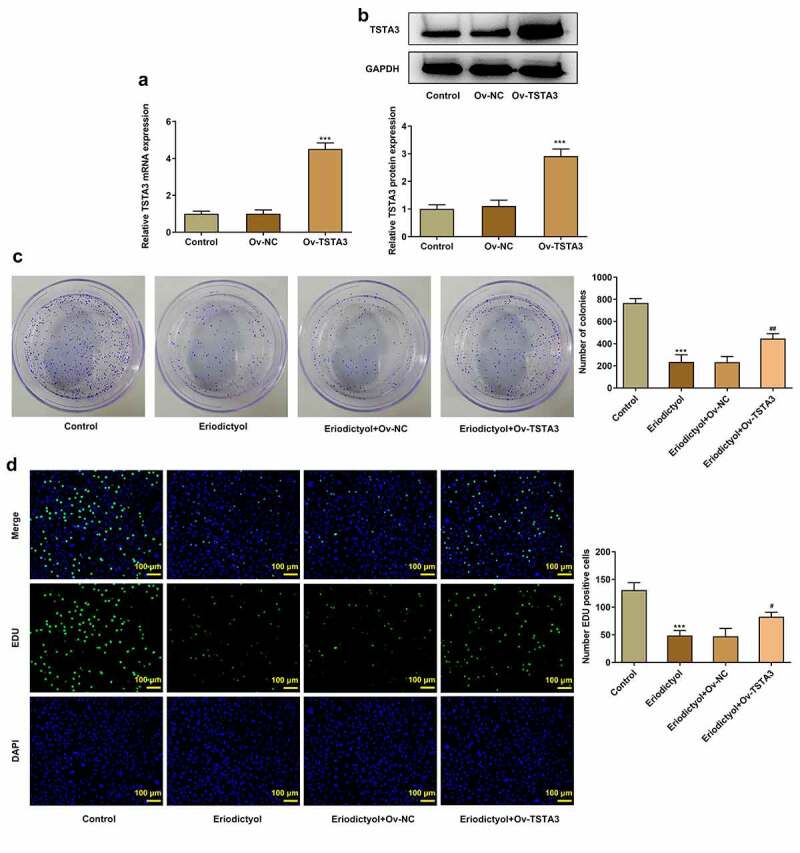
Overexpression of TSTA3 reverses the inhibitory effects of eriodictyol on the clone-forming and proliferative abilities of CRC cells. (a, b) HCT116 cells were transfected with Ov-TSTA3 or Ov-NC and the overexpression efficiency was validated by performing RT-qPCR and Western blot analysis. *** p < 0.001 versus Ov-NC. (c) HCT116 cells receiving eriodictyol treatment were transfected with Ov-TSTA3 or Ov-NC. Colony-formation assay was employed to detect the clone-forming ability of CRC cells. (d) HCT116 cells receiving eriodictyol treatment were transfected with Ov-TSTA3 or Ov-NC. EdU assay was employed to detect CRC cell proliferation. *** p < 0.001 versus Control; ^#^ P < 0.05, ^##^ P < 0.01 versus Eriodictyol + Ov-NC.

In order to expound the biological role of TSTA3 in CRC, HCT116 cells were transfected with Ov-TSTA3, and the overexpression efficiency was validated by performing RT-qPCR and Western blot analysis. Ov-TSTA3 significantly upregulated the mRNA and protein levels of TSTA3 in HCT116 cells (Figure 4(a, b)). Eriodictyol treatment inhibited clone formation and proliferation of HCT116 cells, which were abolished by TSTA3 overexpression (Figure 4(c, d)). We conclude that eriodictyol may diminish the clone-forming and proliferative abilities of CRC cells by downregulating TSTA3 expression.

### Overexpression of TSTA3 reverses the inhibitory effects of eriodictyol on migration, invasion, and EMT of CRC cells

**Figure 5.**
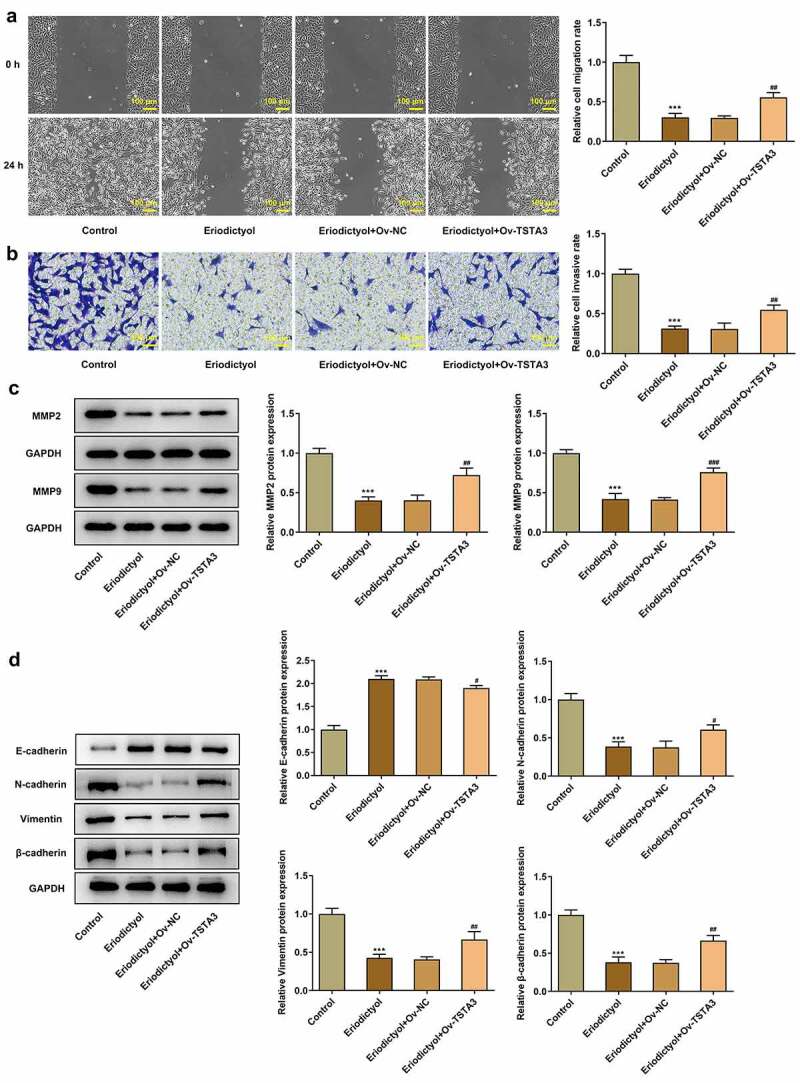
Overexpression of TSTA3 reverses the inhibitory effects of eriodictyol on migration, invasion, and EMT of CRC cells. HCT116 cells receiving eriodictyol treatment were transfected with Ov-TSTA3 or Ov-NC. (a) Wound healing assay was employed to detect CRC cell migration. (b) Transwell invasion assay was employed to detect CRC cell invasion. (c) Western blot analysis was employed to detect the levels of MMP2 and MMP9 in CRC cells. (d) Western blot analysis was employed to detect the levels of E-cadherin, N-cadherin, Vimentin, and β-cadherin in CRC cells. *** p < 0.001 versus Control; ^#^ P < 0.05, ^##^ P < 0.01, ^###^ P < 0.001 versus Eriodictyol + Ov-NC.

Eriodictyol treatment suppressed migration and invasion of HCT116 cells, which were abrogated by TSTA3 overexpression (Figure 5(a, b)). Besides, elevated expressions of MMP2 and MMP9 following transfection of Ov-TSTA3 consistently proved the findings above (Figure 5(c)). Moreover, decreased E-cadherin expression and increased expressions of N-cadherin, Vimentin, and β-cadherin upon transfection of Ov-TSTA3 indicated that upregulation of TSTA3 reversed the suppressing effects of eriodictyol on EMT process in HCT116 cells (Figure 5(d)). To sum up, eriodictyol may repress migration, migration, and EMT of CRC cells by downregulating TSTA3 expression.

### Eriodictyol treatment inhibits the clone-forming and proliferative abilities of CRC cells by downregulating TSTA3 expression to restrain fucosylation

**Figure 6.**
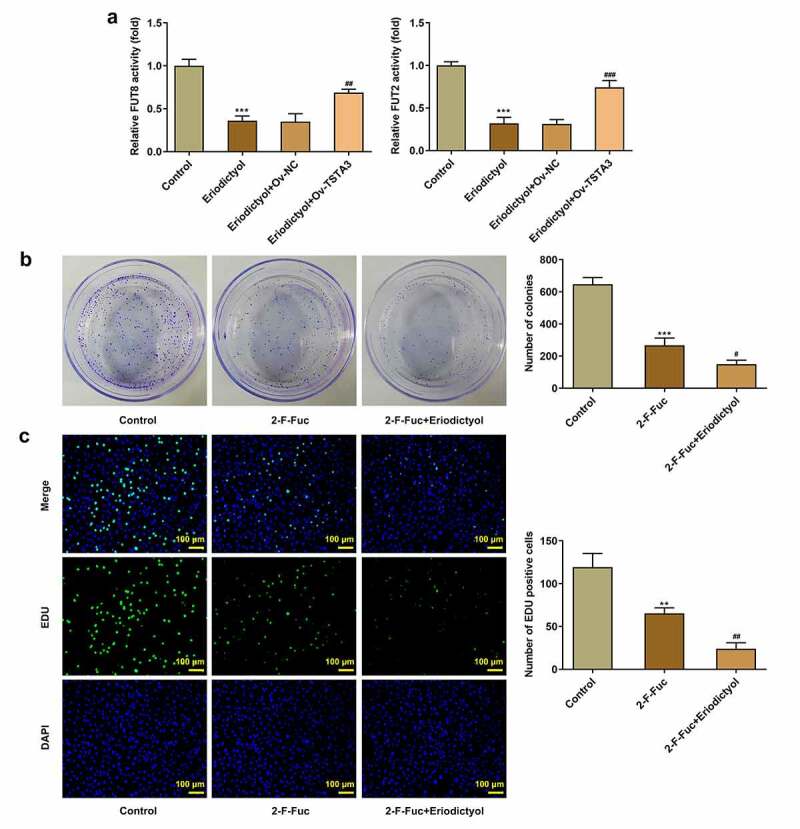
Eriodictyol treatment inhibits the clone-forming and proliferative abilities of CRC cells by downregulating TSTA3 expression to restrain fucosylation. (a) HCT116 cells receiving eriodictyol treatment were transfected with Ov-TSTA3 or Ov-NC. FUT2 and FUT8 enzyme activities were measured using the Glycosyltransferase activity kit. *** p < 0.001 versus Control; ^##^ P < 0.01, ^###^ P < 0.001 versus Eriodictyol + Ov-NC. (b) HCT116 cells were treated with 2-F-Fuc or co-treated with 2-F-Fuc and eriodictyol. Colony-formation assay was employed to detect the clone-forming ability of CRC cells. (c) HCT116 cells were treated with 2-F-Fuc or co-treated with 2-F-Fuc and eriodictyol. EdU assay was employed to detect CRC cell proliferation. ** p < 0.01, *** p < 0.001 versus Control; ^#^ P < 0.05, ^##^ P < 0.01 versus 2-F-Fuc.

In order to probe into the underlying mechanism, we focused on the correlation among eriodictyol, TSTA3, and fucosylation in the development of CRC. It was discovered that eriodictyol treatment decreased the activities of FUT8 and FUT2, which were reversed by the TSTA3 overexpression (Figure 6(a)). Results above suggest that eriodictyol may suppress fucosylation by downregulating the TSTA3 expression. Furthermore, it was demonstrated that fucosylation inhibitor (2-F-Fuc) distinctly inhibited clone formation and proliferation of HCT116 cells, which were reinforced by eriodictyol (Figure 6(b, c)). Overall, eriodictyol may diminish the clone-forming and proliferative abilities of CRC cells by downregulating TSTA3 expression to restrain fucosylation.

### Eriodictyol treatment suppresses migration, invasion, and EMT of CRC cells by downregulating TSTA3 expression to restrain fucosylation

**Figure 7.**
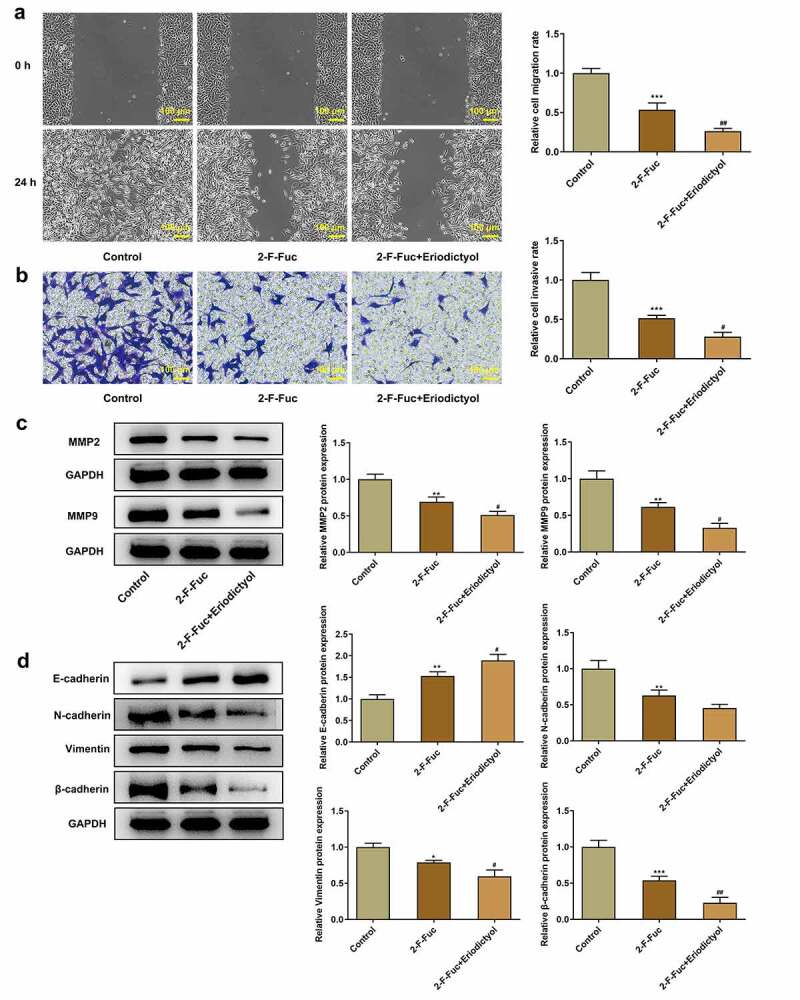
Eriodictyol treatment suppresses migration, invasion, and EMT of CRC cells by downregulating TSTA3 expression to restrain fucosylation. HCT116 cells were treated with 2-F-Fuc or co-treated with 2-F-Fuc and eriodictyol. (a) Wound healing assay was employed to detect CRC cell migration. (b) Transwell invasion assay was employed to detect CRC cell invasion. (c) Western blot analysis was employed to detect the levels of MMP2 and MMP9 in CRC cells. (d) Western blot analysis was employed to detect the levels of E-cadherin, N-cadherin, Vimentin, and β-cadherin in CRC cells. * p < 0.05, ** p < 0.01, *** p < 0.001 versus Control; ^#^ P < 0.05, ^##^ P < 0.01 versus 2-F-Fuc.

Moreover, the 2-F-Fuc diminished the migrative and invasive properties of HCT116 cells, which were strengthened by eriodictyol (Figure 7(a-c)). Besides, eriodictyol treatment further reinforced the inhibitory effects of 2-F-Fuc on EMT in CRC cells (Figure 7(d)). In general, eriodictyol may diminish the migrative and invasive abilities of CRC cells as well as repress the EMT process by downregulating TSTA3 expression to restrain fucosylation.

## Discussion

CRC generally does not show obvious symptoms until the mid-to-late stage or distant metastasis of the disease, so it is easy to be ignored [24]. Hence, exploring related genes in the development of CRC and developing effective therapeutic drugs and important links that affect the biological characteristics of CRC are critical ways to understand the malignancy of tumors and to improve the survival and prognosis of CRC patients.

As a natural flavonoid compound, eriodictyol has been proven to possess anti-tumor effects. It has been reported that eriodictyol exerts inhibitory effects on cell proliferation, migration, as well as invasion of glioma cells via regulating PI3K/Akt/NF-κB signaling pathway [12]. Eriodictyol could inhibit the growth of lung cancer cell lines through induction of mitochondrial-mediated apoptosis, G2/M cell cycle arrest, and inhibition of mTOR/PI3K/Akt cascade signaling pathway [25]. Besides, eriodictyol can exert anti-cancer activity against nasopharyngeal cancer cells by blocking MEK/ERK signaling pathway, inducing cellular autophagy and suppressing cell migration and invasiveness. In the current work, it was confirmed that eriodictyol treatment inhibited the viability, clone-formation, proliferation, migration, invasion, and EMT of CRC cells.

Glycosylation, one of the most common types of protein post-translational modifications [14], is indispensable for more than half of the proteins in the organism to perform their respective functions [26]. Recently, numerous research studies have confirmed that flavonoids can function by regulating protein glycosylation. Ma et al. [27] proved that isoquercitrin could inhibit β-lactoglobulin glycation. Liu et al. [28] indicated that the plant flavonoid luteolin could reduce mucin-type O-glycosylation of the amyloid precursor protein. Abnormal glycosylation is a key factor of tumor malignant transformation and is closely related to the biological behaviors of tumor cells, such as growth, proliferation, migration, and invasion [29,30]. As a common glycosylation modification, fucosylation is involved in lots of malignant transformation events of cancers [31,32]. TSTA3 is one of the key enzymes that is involved in the de novo synthesis and can convert cellular GDP-D-mannose into GDP-L-fucose [28]. It was predicted on the STITCH database that eriodictyol interacted with TSTA3. Importantly, it has been demonstrated that TSTA3 possesses a potent tumor-promoting activity in the regulation of a variety of tumors [33,34]. In the present research, it was discovered that eriodictyol treatment reduced TSTA3 expression in CRC cells. The overexpression of TSTA3 reversed the inhibitory effects of eriodictyol on clone formation, proliferation, migration, invasion, and EMT process of CRC cells. Moreover, eriodictyol suppresses fucosylation by downregulating the TSTA3 expression. Results confirmed that fucosylation inhibitor (2-F-Fuc) inhibited clone formation, proliferation, migration, invasion, as well as EMT of CRC cells, and eriodictyol treatment further reinforced the inhibitory effects of 2-F-Fuc on the malignant behavior of CRC cells.

## Conclusion

In summary, it was revealed that eriodictyol treatment suppressed the clone-forming, proliferative, migratory and invasive abilities of CRC cells as well as repressed EMT process by downregulating TSTA3 expression to restrain fucosylation. The findings above might help to develop promising agents and effective therapeutic targets for CRC therapy in clinic. More importantly, the in-depth mechanism should be further investigated. Still, clinical analysis should be studied in the future to support the findings of this work and to excavate the predictive values of eriodictyol.

## Data Availability

Datasets analyzed during the current study are available from the corresponding author on reasonable request.

## References

[1] Arnold M, Sierra MS, Laversanne M, et al. Global patterns and trends in colorectal cancer incidence and mortality. Gut. 2017;66(4):683–691. 26818619 10.1136/gutjnl-2015-310912

[2] Siegel RL, Miller KD, Goding Sauer A, et al. Colorectal cancer statistics, 2020. CA Cancer J Clin. 2020;70(3):145–164. 32133645 10.3322/caac.21601

[3] Sung H, Ferlay J, Siegel RL, et al. Global cancer statistics 2020: GLOBOCAN estimates of incidence and mortality worldwide for 36 cancers in 185 countries. CA Cancer J Clin. 2021;71(3):209–249. 33538338 10.3322/caac.21660

[4] Qian CN, Mei Y, Zhang J. Cancer metastasis: issues and challenges. Chin J Cancer. 2017;36(1):38. 28372569 10.1186/s40880-017-0206-7 PMC5379757

[5] Li ZN, Zhao L, Yu LF, et al. BRAF and KRAS mutations in metastatic colorectal cancer: future perspectives for personalized therapy. Gastroenterol Rep (Oxf). 2020;8(3):192–205. 32665851 10.1093/gastro/goaa022 PMC7333923

[6] Qadir MI. Role of green tea flavonoids and other related contents in cancer prevention. Crit Rev Eukaryot Gene Expr. 2017;27(2):163–171. 28845765 10.1615/CritRevEukaryotGeneExpr.2017019493

[7] Yan YB, Tian Q, Zhang JF, et al. Antitumor effects and molecular mechanisms of action of natural products in ovarian cancer. Oncol Lett. 2020;20(5):141. 32934709 10.3892/ol.2020.12001 PMC7471673

[8] Imran M, Rauf A, Abu-Izneid T, et al. Luteolin, a flavonoid, as an anticancer agent: a review. Biomed Pharmacother. 2019;112:108612. 30798142 10.1016/j.biopha.2019.108612

[9] Rauf A, Imran M, Khan IA, et al. Anticancer potential of quercetin: a comprehensive review. Phytother Res. 2018;32(11):2109–2130. 30039547 10.1002/ptr.6155

[10] Islam A, Islam MS, Rahman MK, et al. The pharmacological and biological roles of eriodictyol. Arch Pharm Res. 2020;43(6):582–592. 32594426 10.1007/s12272-020-01243-0

[11] Wang Y, Chen Y, Chen Y, et al. Eriodictyol inhibits IL-1β-induced inflammatory response in human osteoarthritis chondrocytes. Biomed Pharmacother. 2018;107:1128–1134. 30257325 10.1016/j.biopha.2018.08.103

[12] Li W, Du Q, Li X, et al. Eriodictyol inhibits proliferation, metastasis and induces apoptosis of glioma cells via PI3K/Akt/NF-κB signaling pathway. Front Pharmacol. 2020;11:114. 32158391 10.3389/fphar.2020.00114 PMC7052300

[13] Tang L, Qin Y, Ling K, et al. Eriodictyol inhibits the growth of CNE1 human nasopharyngeal cancer growth by targeting MEK/ERK signalling pathway, inducing cellular autophagy and inhibition of cell migration and invasion. J BUON. 2020;25(5):2389–2394. 33277860

[14] Taniguchi N, Kizuka Y. Glycans and cancer: role of N-glycans in cancer biomarker, progression and metastasis, and therapeutics. Adv Cancer Res. 2015;126:11–51. 25727145 10.1016/bs.acr.2014.11.001

[15] Cascio S, Finn OJ. Intra- and extra-cellular events related to altered glycosylation of MUC1 promote chronic inflammation, tumor progression, invasion, and metastasis. Biomolecules. 2016;6(4):39. 10.3390/biom6040039 PMC5197949 27754373

[16] Du N, Song L, Li Y, et al. Phytoestrogens protect joints in collagen induced arthritis by increasing IgG glycosylation and reducing osteoclast activation. Int Immunopharmacol. 2020;83:106387. 32172207 10.1016/j.intimp.2020.106387

[17] Hao S, Fan Q, Bai Y, et al. Core fucosylation of intestinal epithelial cells protects against salmonella typhi infection via up-regulating the biological antagonism of intestinal microbiota. Front Microbiol. 2020;11:1097. 32528455 10.3389/fmicb.2020.01097 PMC7266941

[18] Shan M, Yang D, Dou H, et al. Fucosylation in cancer biology and its clinical applications. Prog Mol Biol Transl Sci. 2019;162:93–119. 30905466 10.1016/bs.pmbts.2019.01.002

[19] Iwona R, Katarzyna S. Glycosylation of proteins of human skin fibroblasts is changed by rosmarinic acid. Naunyn Schmiedebergs Arch Pharmacol. 2020;393(3):419–427. 31654087 10.1007/s00210-019-01732-0

[20] Gao Y, Zhang G, Liu J, et al. Tissue-specific transplantation antigen P35B functions as an oncogene and is regulated by microRNA-125a-5p in lung cancer. Oncol Rep. 2021;45(5):72. 33760213 10.3892/or.2021.8023 PMC8020207

[21] Yang J, Kong P, Yang J, et al. High TSTA3 expression as a candidate biomarker for poor prognosis of patients with ESCC. Technol Cancer Res Treat. 2018;17:1533033818781405. 29950151 10.1177/1533033818781405 PMC6048620

[22] Sun Y, Liu X, Zhang Q, et al. Oncogenic potential of TSTA3 in breast cancer and its regulation by the tumor suppressors miR-125a-5p and miR-125b. Tumour Biol. 2016;37(4):4963–4972. 26531722 10.1007/s13277-015-4178-4

[23] Livak KJ, Schmittgen TD. Analysis of relative gene expression data using real-time quantitative PCR and the 2(-Delta Delta C(T)) method. Methods. 2001;25(4):402–408. 11846609 10.1006/meth.2001.1262

[24] Tseng JY, Yang CY, Liang SC, et al. Interleukin-17A modulates circulating tumor cells in tumor draining vein of colorectal cancers and affects metastases. Clin Cancer Res. 2014;20(11):2885–2897. 24677375 10.1158/1078-0432.CCR-13-2162

[25] Zhang Y, Zhang R, Ni H. Eriodictyol exerts potent anticancer activity against A549 human lung cancer cell line by inducing mitochondrial-mediated apoptosis, G2/M cell cycle arrest and inhibition of m-TOR/PI3K/Akt signalling pathway. Arch Med Sci. 2020;16(2):446–452. 32190156 10.5114/aoms.2019.85152 PMC7069446

[26] Veillon L, Fakih C, Abou-El-Hassan H, et al. Glycosylation changes in brain cancer. ACS Chem Neurosci. 2018;9(1):51–72. 28982002 10.1021/acschemneuro.7b00271 PMC5771830

[27] Ma TX, Zhang L, Xu L, et al. Mitigation of isoquercitrin on β-lactoglobulin glycation: insight into the mechanisms by mass spectrometry and interaction analysis. Int J Biol Macromol. 2020;155:1133–1141. 31715232 10.1016/j.ijbiomac.2019.11.080

[28] Liu F, Xu K, Xu Z, et al. The small molecule luteolin inhibits N-acetyl-α-galactosaminyltransferases and reduces mucin-type O-glycosylation of amyloid precursor protein. J Biol Chem. 2017;292(52):21304–21319. 29061849 10.1074/jbc.M117.814202 PMC5766936

[29] Fang R, Xu F, Shi H, et al. LAMTOR5 raises abnormal initiation of O-glycosylation in breast cancer metastasis via modulating GALNT1 activity. Oncogene. 2020;39(11):2290–2304. 31836847 10.1038/s41388-019-1146-2

[30] Liu HM, Ma LL, Cao B, et al. Progress in research into the role of abnormal glycosylation modification in tumor immunity. Immunol Lett. 2021;229:8–17. 33186635 10.1016/j.imlet.2020.11.003

[31] Bastian K, Scott E, Elliott DJ, et al. FUT8 alpha-(1,6)-fucosyltransferase in cancer. Int J Mol Sci. 2021;22(1):455. 10.3390/ijms22010455 PMC7795606 33466384

[32] Xu J, Xiao Y, Liu B, et al. Exosomal MALAT1 sponges miR-26a/26b to promote the invasion and metastasis of colorectal cancer via FUT4 enhanced fucosylation and PI3K/Akt pathway. J Exp Clin Cancer Res. 2020;39(1):54. 32209115 10.1186/s13046-020-01562-6 PMC7092616

[33] Kizuka Y, Nakano M, Yamaguchi Y, et al. An alkynyl-fucose halts hepatoma cell migration and invasion by inhibiting GDP-fucose-synthesizing enzyme FX, TSTA3. Cell Chem Biol. 2017;24(12):1467–1478.e5. 29033318 10.1016/j.chembiol.2017.08.023

[34] Zhang L, Gao Y, Zhang X, et al. TSTA3 facilitates esophageal squamous cell carcinoma progression through regulating fucosylation of LAMP2 and ERBB2. Theranostics. 2020;10(24):11339–11358. 33042286 10.7150/thno.48225 PMC7532669

